# Biodiversity Assessment in Incomplete Inventories: Leaf Litter Ant Communities in Several Types of Bornean Rain Forest

**DOI:** 10.1371/journal.pone.0040729

**Published:** 2012-07-16

**Authors:** Martin Pfeiffer, Dirk Mezger

**Affiliations:** 1 Department of Ecology, National University of Mongolia, Ulaanbaatar, Mongolia; 2 Institute of Experimental Ecology, University of Ulm, Ulm, Germany; Northwestern University, United States of America

## Abstract

Biodiversity assessment of tropical taxa is hampered by their tremendous richness, which leads to large numbers of singletons and incomplete inventories in survey studies. Species estimators can be used for assessment of alpha diversity, but calculation of beta diversity is hampered by pseudo-turnover of species in undersampled plots. To assess the impact of unseen species, we investigated different methods, including an unbiased estimator of Shannon beta diversity that was compared to biased calculations. We studied alpha and beta diversity of a diverse ground ant assemblage from the Southeast Asian island of Borneo in different types of tropical forest: diperocarp forest, alluvial forest, limestone forest and heath forests. Forests varied in plant composition, geology, flooding regimes and other environmental parameters. We tested whether forest types differed in species composition and if species turnover was a function of the distance between plots at different spatial scales. As pseudo-turnover may bias beta diversity we hypothesized a large effect of unseen species reducing beta diversity. We sampled 206 ant species (25% singletons) from ten subfamilies and 55 genera. Diversity partitioning among the four forest types revealed that whereas alpha species richness and alpha Shannon diversity were significantly smaller than expected, beta-diversity for both measurements was significantly higher than expected by chance. This result was confirmed when we used the unbiased estimation of Shannon diversity: while alpha diversity was much higher, beta diversity differed only slightly from biased calculations. Beta diversity as measured with the Chao-Sørensen or Morisita-Horn Index correlated with distance between transects and between sample points, indicating a distance decay of similarity between communities. We conclude that habitat heterogeneity has a high influence on ant diversity and species turnover in tropical sites and that unseen species may have only little impact on calculation of Shannon beta diversity when sampling effort has been high.

## Introduction

Biodiversity research on tropical insect communities is hampered by different factors. The extreme species-richness of many tropical taxa inevitably leads to sampling large numbers of singleton species, steep rarefaction curves, and incomplete inventories [1]. For alpha-diversity there is a long history of methods to overcome the problem of undersampling, e.g. by species richness estimators and unbiased species diversity indices [2]–[4]. When evaluating beta diversity incomplete sampling may lead to pseudo-turnover of species (i.e. an inflation of true turnover rates due to species actually present, but missed in an -incomplete- sampling) [5]. Due to the high numbers of rare species pseudo-turnover will be a frequent problem for calculating beta diversity in tropical communities and few attempts have been made to assess beta diversity in specious tropical insects [6], [7]. Chao and coworkers have developed several methods to estimate similarity of species communities, which are based on the Morisita and Soerensen Indices [8], [9]. The whole methodology of diversity calculation has recently been strongly influenced by the work of Jost [10], [11] and our paper is fully based on his concepts and terminology.

Partitioning of species gamma diversity into its alpha and beta components [11] provides helpful insights into diversity patterns across landscapes that can be used for species conservation [12]–[14] – a perspective that is especially important for tropical communities faced with increasing socioeconomic pressures [15]. Until recently, however, this method has been applied only in temperate ecosystems with complete species records [13]. Woodcock and coworkers [14] were the first to apply diversity partitioning of ant communities in tropical forests, and they carefully compared results with and without rare species. Inclusion or exclusion of rare species can have significant impact on the conclusions we draw from biodiversity results, especially when primary and degraded habitats are compared [16]. Rare species in primary forests provide specific ecological functions [17] and are maintained by ecosystem processes [18]. Thus, a comparison among highly diverse primary forest patches must account for them and ought to include potentially rarer, unseen species. A solution for this problem has been offered by Marcon *et al.* [19], who developed a self-contained definition of β-entropy and a bias correction for its estimator, thereby enabling bias-corrected diversity partitioning of Shannon diversity in highly diverse communities.

**Figure 1:**
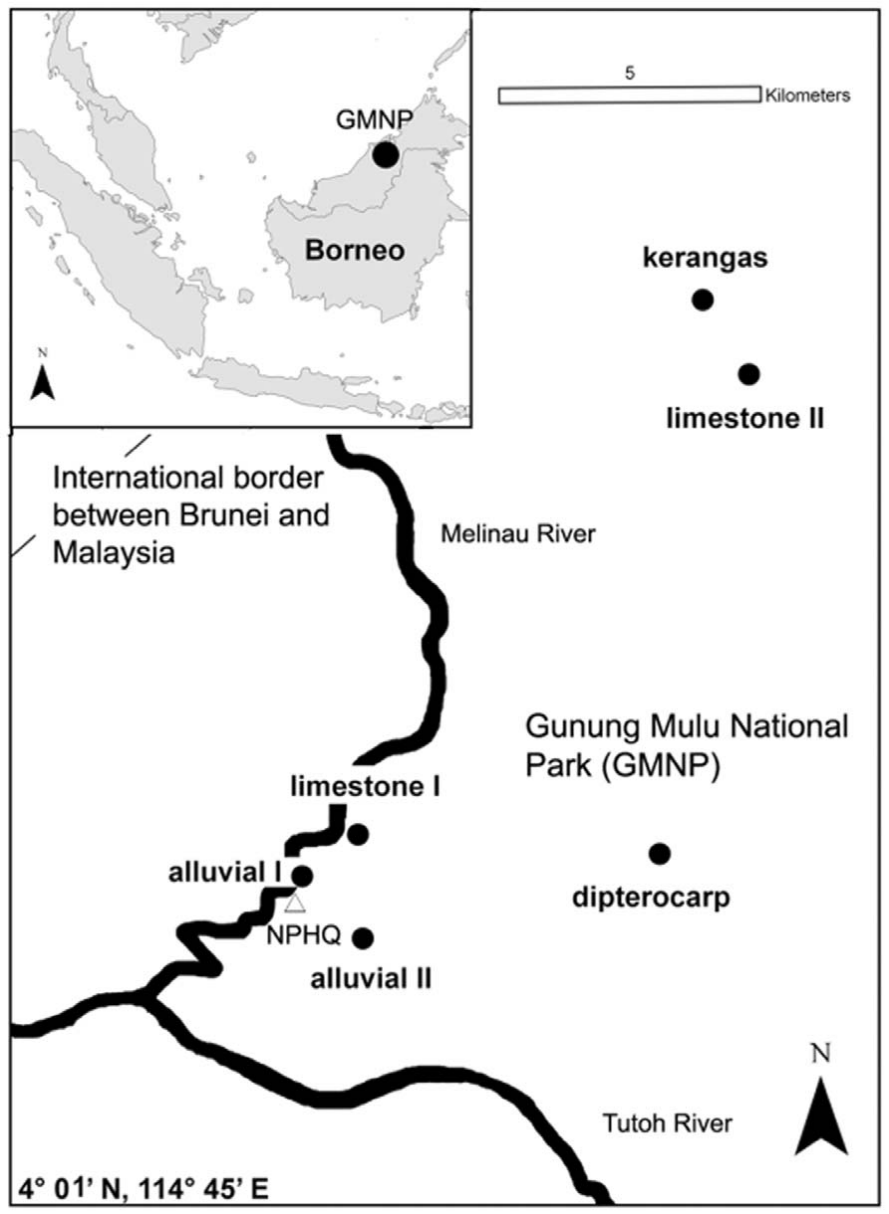
Location of the six transects in Gunung Mulu National Park. The position of the National Park’s headquarter (HQ) is marked with a white triangle. The coordinates on the map are at the lower-left corner. The inserted map shows the position of this protected area on Borneo.

We aimed at exploring the effects of unseen species on biodiversity partitioning and used this unbiased estimator, as well as traditional partitioning, to assess diversity of an extremely diverse tropical ground ant assemblage from the Southeast Asian island of Borneo. There are currently 717 species and 52 additional subspecies described from Borneo [20]. High species richness and alpha diversity have been reported for arboreal species assemblages [21] and leaf litter ants [14], [22]. Our long-term research in four forest habitats of the Gunung Mulu National Park (GMNP) has demonstrated that local leaf litter ant communities are structured by niche differences and neutral mechanisms [23]. However, a detailed analysis of the diversity pattern of ant communities in the highly heterogeneous habitats at GMNP (Figure 1) is still missing.

Little is known about beta-diversity in tropical ant species. Vasconcelos *et al.* [24] reported “moderate” species turnover of ants along a 2000 km transect through alluvial forests in the Amazon river basin, while Woodcock *et al.* [14], who worked in northern Borneo, reported “high” beta diversity between their plots, especially in logged forest. Here, for the first time, we compare ant diversity in different types of tropical forest: diperocarp forest, alluvial forest, limestone forest and Kerangas (heath forest) and apply various statistics to account for potential incomplete data. Depending on former results that stressed certain species’ distinct habitat choice [23] and theoretical considerations [5], we expected.

1. a high species turnover between the ant communities of these different habitats.
2. a remarkable reduction of beta diversity after accounting for unseen species as pseudo-turnover may bias beta diversity.

## Results

In total we found 206 ant species in all of the four forest types. They represented ten subfamilies and 55 genera. We recorded one to 24 species per genus, including two genera with more than twenty species (*Pheidole* and *Strumigenys*). Thirteen further genera contained between five and nine species. We sampled 23 ant genera represented by only one species. The most common species were *Strumigenys rofocala* (76 occurrences), *Monomorium* sp. 1 (69 occurrences), *Hypoponera* sp. AL16B (61 occurrences) and *Oligomyrmex* sp. 2 (50 occurrences). 25% of all species were only collected with a single occurrence. A full list of all species is given in the Table S1.

**Figure 2:**
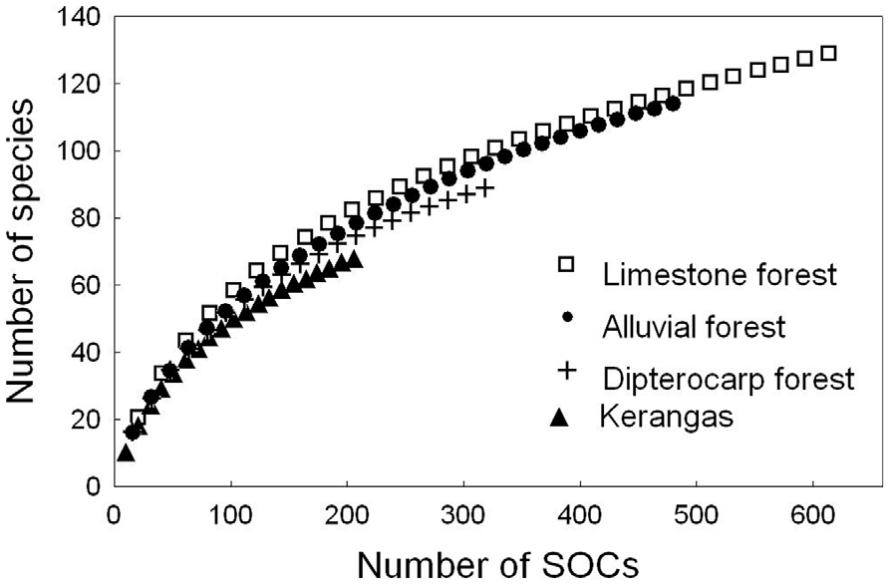
Mao Tao rarefaction curves of all four forest types. We sampled 20 points in dipterocarp forest and Kerangas and 30 points in limestone and alluvial forest. Calculation was based on species occurrences (SOCs).

In 20 samples from each forest type we found 68 species of ants in Kerangas, 89 species in dipterocarp forest, 96 species in alluvial forest, and 110 species in limestone forest. In 30 samples from alluvial and limestone forest we detected 114 and 129 species, respectively (Table 1, Figure 2). Mean species densities (ANOVA F_3,96_ = 25.9, p<0.001) and ant individuals density (ANOVA F_3,96_ = 4.1, p<0.01) differed significantly between the forest types (Table 1). These two densities were significantly correlated (Pearson correlation: r^2^ = 0.27; t = 6.07; p<0.0001).

**Table 1:** Sample parameters, collected and estimated numbers of ant species in the four forest types of Gunung Mulu National Park.

Forest type	Alluvial	Limestone	Dipterocarp	Kerangas	All types
Samples	30	30	20	20	100
Ant density [individuals/m^2^]	378 (±329)	451 (±252)	378 (±316)	154 (±142)	355 (±266)
Total species occurrences	480	614	319	206	1619
Species number	114	129	89	68	206
Species density [species/m^2^]	17 (±5.2)	22 (±5.2)	16 (±3.7)	10 (±3.8)	16 (±5.6)
% of species collected	72.4	61.6	63.5	59.7	79.2
Estimator (acc. Brose et. al 2004)	Jackknife 1	Jackknife 2	Jackknife 2	Jackknife 2	Jackknife 1
Estimated species	157	210	140	114	260

Given are sample size, ant density, species occurrences, the collected species number and density, the sample completeness and the species estimator chosen after [40], as well as the number of estimated species. **±** SD in round parentheses.

Species richness in all forest types was estimated at 260 species, representing a total sampling coverage of 79% (estimated using Jackknife 1). Based on several species estimators, we found between 60% and 72% of all species of the respective forests (Table 1). In all cases sample completeness was >0.5, and thus justified the use of unbiased species diversity estimators (see methods section below).

Shannon entropy H_α_ and the resulting effective species numbers, sensu Jost [10], D_α_, demonstrated that alpha diversity was highest in limestone forest, with lower diversity occurring in alluvial and dipterocarp forest, while the Kerangas included the lowest ant diversity (Table 2).

**Table 2:** Alpha diversity measures at different sample size for the four forest types.

Diversity measure	Common sample size	Alluvial forest	Limestone forest	Dipterocarp forest	Kerangas
Shannon entropy Hα	206 SOCs	4.0±0.07	4.13±0.06	3.98±0.04	3.86±0.02
Shannon diversity Dα	206 SOCs	54.36	62.18	53.52	47.47
Shannon entropy Hα	470 SOCs	4.18±0.01	4.30±0.03	–	–
Shannon diversity Dα	470 SOCs	65.37	73.7	–	–
Simpson diversity	206 SOCs	50.56	63.72	50.64	41.56

Given are Shannon entropy and the resulting effective species numbers - Shannon diversity sensu Jost [10]- for all forest types at a common sample size of 206 SOCs and for Alluvial and limestone forest separately at a common sample size of 470 SOCs. Additionally, Simpson diversity at 206 SOCs is calculated for all forest types. These results are not corrected for unseen species.

Partitioning of the diversity among the four forest types on the basis of all weighted samples revealed that alpha species richness and alpha Shannon diversity were significantly smaller than expected, and that beta-diversity for both measurements was significantly higher than expected by chance (Table 3).

**Table 3:** Hierarchical multiplicative partitioning of α and β components for species richness and Shannon diversity *D* compared to expected values from 1000 randomizations with PARTITION V3.

All foresttypes	Species richness			Shannon diversity D		
Diversity	Observed	Expected	P	Observed	Expected	P
α	109.76	126.71	<0.0001	67.90	81.58	<0.0001
β	1.88	1.63	<0.0001	1.48	1.23	<0.0001
γ	206.34	206.53		100.49	100.34	

For calculation of Shannon diversity samples could be weighted according their abundances.

The respective values for the biased corrected estimations of the effective numbers of species *D_α_, which included unseen species, are much higher and given in Table 4 (the corresponding values for *H_α_, *H_β_ and *H_γ_ are listed in Table S2). Estimates of beta-diversity, the unbiased effective numbers of communities *D_β_, ranged between 1.27 to 1.84 for the four forest types and resulted in a weight sum *D_β_ of 1.42 (Table 4), which differed only slightly from the biased result. This value is highly significant, as the 95% confidence intervals show that the probability to have *D_β_ = 0 is so low that it can be considered as impossible. Moreover, the lower 95% confidence interval of 1.38 was well larger than the expected value of beta diversity, which was 1.23 as estimated with null models (see Table 3). Thus both, calculated Shannon beta diversity and the unbiased estimation of Shannon beta diversity, were higher than expected. As the program of Marcon *et al.* [19] did not calculate 95% confidence intervals for alpha diversity, we could not prove this for the unbiased estimation of Shannon alpha diversity.

**Table 4:** Unbiased estimations of alpha, beta and gamma Shannon diversity*D according to the partitioning procedure of Marcon *et al.* [19].

Forest type	Alluvial	Limestone	Dipterocarp	Kerangas	All forests
Unbiased alpha Shannon diversity *D_α_	78.84	87.45	71.12	59.35	77.50
Unbiased beta Shannon diversity *D_β_	1.38	1.27	1.57	1.84	1.42 [1.38; 1.47]
Unbiased Shannon gamma diversity *D_γ_					110.52

Given are the unbiased estimators for alpha and beta Shannon diversity for the single forest types; for all forests their weighted sums are given, as well as the resulting unbiased gamma Shannon diversity. These values have been calculated by additive partitioning of the Shannon entropy H; the calculation is given in Table S2. Upper and lower 95% confidence interval for the estimator of Shannon beta diversity in squared brackets, range of *D_β_: 1.0–4.0.

**Figure 3:**
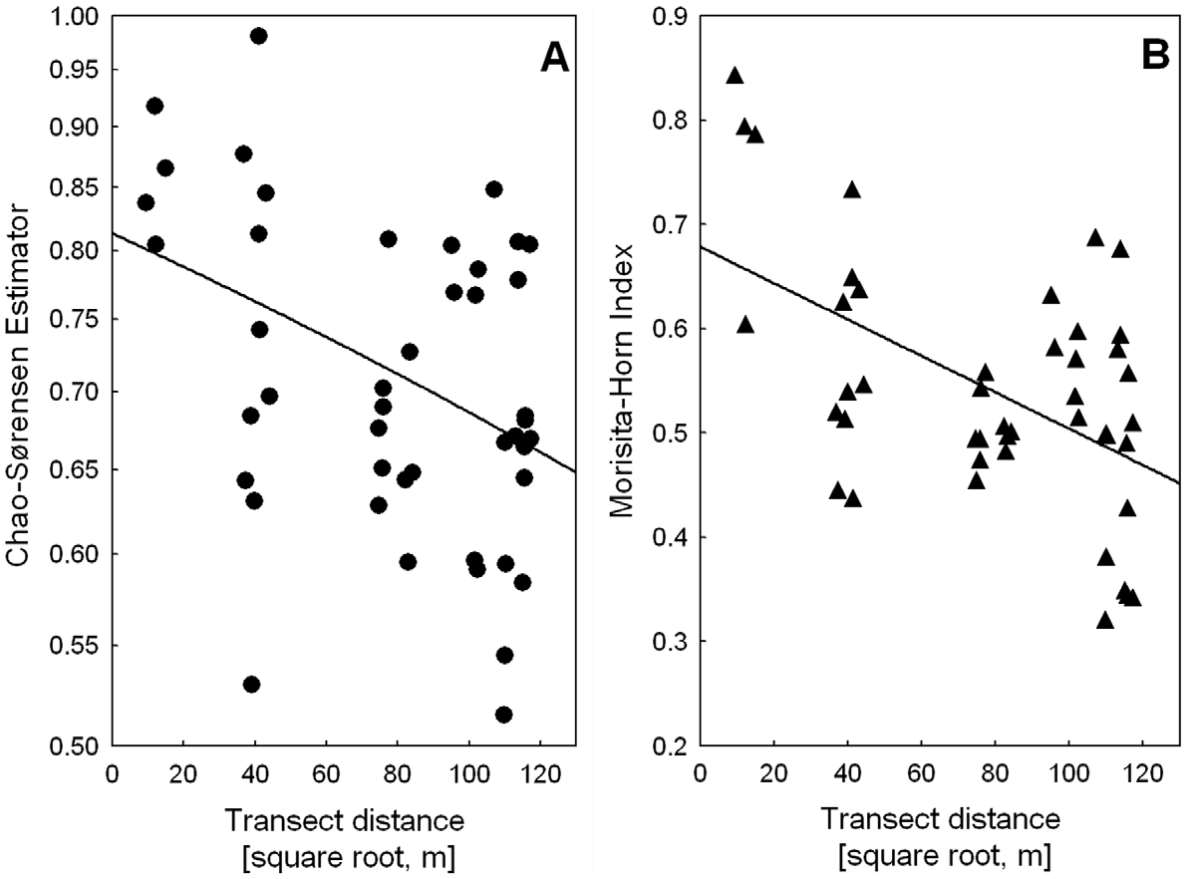
Transect distances and beta diversity measures. Statistic calculations were done with mantel tests on inverted indices, regressions lines are only given for visualization. Mind the different scales of the Y-axes. 3a) Distance decay of transect similarity as shown by Chao-Sørensen estimations (Regression line y = 0.8134−0.0013*x). 3b) Distance decay of transect similarity as demonstrated by the Morisita-Horn index (Regression line y = 0.6785−0.0017).

**Figure 4:**
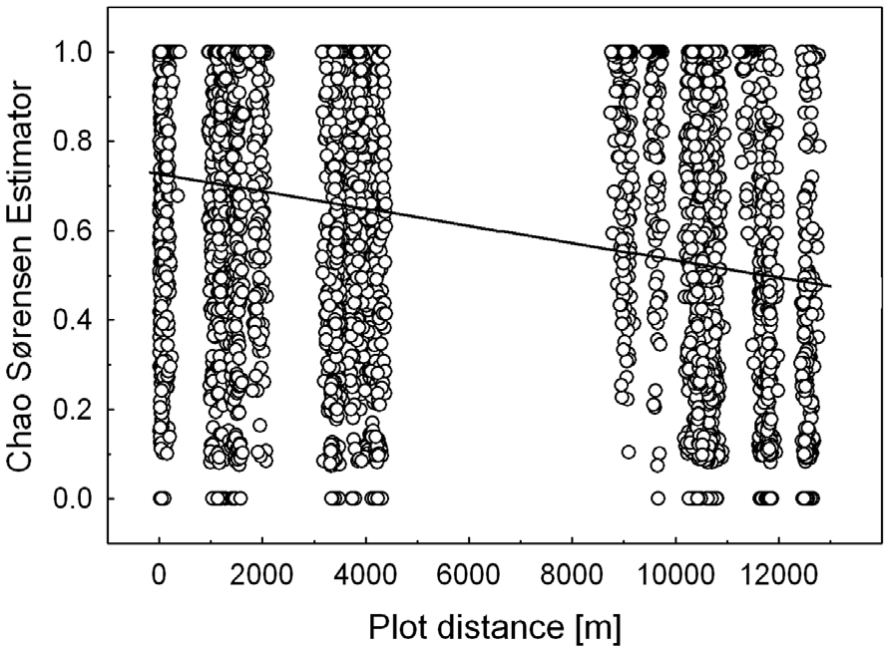
Distance decay of similarity of sampling points demonstrated by Chao Sørensen estimations. Statistic calculation was done with a mantel test and the inverted index (n = 4950), the regression line is only given for visualization (Regression line y = 0.7264−1.9279E-5*x).

The respective *D_β_ values for the weighted comparison of single forest types, although significant as proved by 95% confidence intervals, were between 1.2 and 1.3 for all comparisons, thus demonstrating only a moderate species turnover among the different forest types (Table 5). Beta diversity of single transects (each with 10 samples only) was correlated to transect distance as calculated with the inverted Morisita-Horn index (Mantel correlation with (1-Morsisita-Horn index) n = 45, r = 0.4633, p = 0.013), or with the inverted Chao-Sørensen index (Mantel correlation with (1- Chao-Sørensen index) n = 45, r = 0.3634, p = 0.006)(Figure 3). Inverted Chao-Sørensen indices of single sampling points (n = 4950) were also correlated with distance (Figure 4, Mantel correlation with (1- Chao-Sørensen index) r = 0.2635, p = 0.001), thus demonstrating that dissimilarity of samples rose with the distance between them.

**Table 5:** Unbiased estimated Shannon beta-diversity *D_β_ between the four forest types as calculated after the framework of Marcon *et al.* [19].

	Limestone	Dipterocarp	Kerangas
**Alluvial**	1.27 [1.22; 1.32]	1.25 [1.21; 1.29]	1.24 [0.18; 0.26]
**Kerangas**	1.23 [1.19; 1.27]	1.27 [1.22; 1.32]	–
**Dipterocarp**	1.22 [1.18; 1.26]	–	–

Upper and lower 95% confidence interval in squared brackets, range of *D_β_: 1.0–2.0.

Results are weight according to the number of transects.

## Discussion

We compared four forest types that differed in plant composition, geology, flooding regimes and other environmental parameters [23]. These habitats support discrete ant communities with distinct indicator species [23] and differing temperature niches of ants [25]. Alpha diversity of habitats differed remarkably with estimated species richness (Table 1), which was lowest in Kerangas (114 species), where wet, thick soil layers with low nutrient contents and ph-values occurred, and highest in the limestone forest (210 species), where we found alkaline, nutrient rich conditions in stony, well drained soils with a high structural heterogeneity and dense lower vegetation [23]. These differences were reflected by the densities of individuals and species in these habitats, which were much higher in the nutritious, heterogeneous limestone forest that provided much better nesting and foraging conditions. Interestingly, alluvial forest was found to be species rich, thus confirming that a highly evolved ant community has been able to adapt to the living conditions in this disturbed, periodically short term inundated habitat, e.g. by specialized nest structures [25]. Dipterocarp forest in GMNP proved to be relatively species poor, especially when compared with dipterocarp forest in Danum Valley (DV) in northern Borneo for which 244 and 131 leaf litter ant species have been estimated, respectively [14], [22]. This may partly be due to the much higher sampling effort in these studies; however, dipterocarp forests in GMNP are also potentially less suitable for leaf litter ants because they occur on very poor soils and include tree species and palms of Kerangas forest with scleromorphic leaves [23], [26]. Species density in primary dipterocarp forests was much lower in GMNP than in DV (16.19 vs. 22.73 species) [14].

Shannon alpha diversity calculations corroborated these findings; however, results for the unbiased estimation of Shannon alpha diversity (Table 4) were much higher than those of the standard calculations (Tables 2 and 3). This is astonishing if we take into consideration that unseen species should be less abundant or even rare and thus would have less impact on Shannon diversity, which gives impact to the more abundant species. Actually, however, mean effective numbers of species in single plots were 30% higher in biased than in unbiased calculation, thus reflecting the high values of estimated species richness. This deviation decreased to 11% for the pooled samples of all forest types, demonstrating the effect of comprehensive sampling. The number of singletons in our study was about 40% for each of the forest types, but dropped to 25% when habitats were pooled. At the same time total sampling coverage rose from a mean of 64.3% to a total of 79%. This stresses the importance of large data sets in tropical studies to avoid undersampling [1].

As we had hypothesized, unbiased estimates of Shannon beta diversity were lower than estimates obtained from biased partitioning, but the difference between methods was small, suggesting that pseudo-turnover of species was low, and most unseen species preferred different forest habitats. This clear result demonstrates the usefulness of unbiased Shannon diversity calculation, especially, as at the same time we detected highly biased values for alpha and gamma diversity.

Overall values for beta diversity were relatively low: pairwise comparisons of unbiased Shannon beta diversity between single forest types resulted in relatively low values (ranging potentially between 1 to 2), equaling about 1.2 equally distributed communities. Weighted mean unbiased Shannon beta diversity of all 4 forest types was only 1.4 (potential range from 1 to 4). Although the unequal weights of the plots may account for a part of these low beta diversity values, the main factor is the little weight of singletons in a calculation that accounts for the percentage impact of a single individual, as Shannon diversity does. While at maximum 53% of all the species were sampled in only one forest habitat (the limestone forest), this high species turnover with the other forest types had only little impact on Shannon beta diversity due to the high partition of singletons involved. This is demonstrated when we compare the partitioning results for biased Shannon beta diversity of 1.5 (range 1–4) with the higher beta diversity of species richness of 1.9 (range 1–4), thus pointing to a species turnover of almost 50% (Table 3). However, these figures are not corrected for unseen species and require careful interpretation.

Diversity partitioning proved that beta diversity among forest types was higher than expected and alpha diversity was lower than expected, as shown for biased species richness and Shannon diversity, and corroborated using the unbiased Shannon diversity estimator. This points again towards the divergence of ant niches among forest types that supported differing species. Furthermore beta diversity between transects and single sample points in GMNP as measured by Chao-Sørensen and Morisita-Horn Indices were correlated with pairwise distance separating them, a pattern similar to that described for ants in Amazonia [24] and for ants of arid Iran [13] on much larger scales, however, in more homogenous habitats. Dispersal limitation of ant gynes is generally responsible for this persistent pattern, in the topographically complex GMNP it is reinforced by between habitat diversity. This “ant result” is contrary to that of plant specific insect herbivores that show much less decay of similarity between communities, indicating a lack of dispersal limitation [6].

If species show limited dispersal, mountain ranges and other obstacles may create sufficient opportunity for allopatric speciation. Mountainous regions are rich with species due to species turn-over with altitude [27] and local endemics [28]. These findings demonstrate that topographically complex landscapes are drivers of species diversity in “extra diverse” countries. For hilly Borneo, moths [7], [29] also exhibit high beta-diversity between different areas of the island. High beta-diversity of tropical insect communities has also been demonstrated for phytophagous beetle assemblages in two forest types in Panama, caused by differing precipitation rates in these habitats due to the complex geography of the area [30].

Woodcock et al. [14], reported beta species richness of 1.96 and 2.24 from northern Borneo for pairwise site comparisons of primary and twice logged dipterocarp forests at six sites in Danum Valley. These values are relatively lower than ours, as they have a potential range from 1 to 6 communities. Vasconcelos *et al* found a mean value of 0.62 for the (simple) Chao–Sørensen index of their 26 sampling sites along a 2000 km transect on the large Amazonia river plains [24], comparable to our ten transects sampled on much smaller scale, which had a mean Chao–Sørensen index of 0.59 when recalculated. More standardized research on ant beta diversity at different spatial scales in the tropics is urgently needed to answer the question whether species turnover is usually high or low.

Extrapolating our results to the whole of mountainous Borneo, we conclude that there might be a high species turnover, with many species being restricted to certain areas. Effective conservation measurements for Borneo would require including areas from all parts of this highly heterogeneous island into a conservation network, as it was proposed by the “Heart of Borneo” conservation program [31]. At the same time our study stresses the use of ants as diversity indicators in tropical conservation studies; the effectiveness of ant sampling has proven ants as a reliable tool for estimating seen and unseen diversity and thus as a useful and applicable to conservation monitoring. Finally, we believe that bias corrected biodiversity partitioning will be recognized as an important tool for ecologists to understand the factors that shape biodiversity patterns, especially in highly diverse tropical regions.

## Materials and Methods

### Ethics Statement

The research has been conducted according to the Malaysian law.

### Study Area

The study was conducted in the 528 km^2^ Gunung Mulu National Park (GMNP)(4° 57′N, 114° 47′E) in Sarawak (Malaysia) on Borneo [32], [33]. The climate in the lowlands is tropical, with mean air temperatures of about 26°C and yearly rainfall of 4000–5000 mm (Sarawak Weather Service, per. comm.). All fieldwork took place between 5^st^ April 2006 and 17^th^ October 2007. Four types of lowland forest were sampled: alluvial forest, limestone forest, Kerangas, and dipterocarp forest. Forests differ in soil, slope, flood frequency, and vegetation structure; they range in altitude between 50 and 250 m a.s.l. [26]. None of the research plots were separated by more than 15 km. A detailed description of these forests is given elsewhere [23].

### Sampling

In each of the four forest types, we established two neighboring 200 meter transect lines, each with 10 evenly spaced sampling points, according to the ALL-protocol [34]. Due to the comfortable accessibility of limestone and alluvial forest, we established a third transect line in these forest types. The third alluvial transect was established 2 km away from the first alluvial transect, while the third limestone transect was 14 km away from the first (Figure 1). We used a metal frame 1.0 by 1.0 meter in size to mark sample points and to reduce the numbers of fleeing arthropods during sampling. Leaf litter and soil were collected separately and concentrated by sifting with a metal sieve (mesh size 12 mm). We collected soil up to a depth at which a change in color signaled the end of the top layer. Finally, we recorded the geographic position of each point. As continuous canopy cover made it impossible to locate site positions directly by GPS (Garmin GPS 12 XL), we mapped nearby positions and located original positions from satellite maps in Google Earth^©^ 4.3.

The sieved matter was put into canvas bags for transport, which lasted not longer than one day, and extracted with Winkler-bags. Each sample point and each sampled layer was extracted separately [35]. The high air humidity in GMNP made it necessary to hang up the Winkler-bags in the air-conditioned environment of our lab, where they remained for seven days for drying. Arthropods leaving the soil were collected and stored in 70% ethanol. As a control for extraction efficiency, we checked ten percent of the soil samples for remaining arthropods after processing by intensive visual inspection and found less than 3% remaining ant individuals.

### Taxonomy

Voucher specimens were mounted for all ant species. These specimens are kept in the ‘AntBase.Net Collection’ (ABNC) currently housed at the University of Landau, Germany, with Automontage^©^ photographs of most species being available via http://www.antbase.net. Identification of the ant genera was done using Bolton [36]. For species identification we used the literature cited in Pfeiffer *et al.* [20].

### Calculation of Species Richness and Diversity

To assess sample completeness we computed species rarefaction curves for all forest types [37]. All ants of one (morpho-) species that were found at a sampling point were counted as one species occurrence (SOC). A sample comprised all species collected at a sample point on one square meter in leaf litter and soil. We applied the Mao Tau rarefaction formula [38] to calculate the sample-based rarefaction curves that were plotted with the number of SOCs on the x-axis [39].

We optimized the estimation of species richness by choosing the best estimator with a method suggested by Brose & Martinez [40]. Therefore, we 1) estimated the species richness based on all samples with a range of estimators (ACE, ICE, Chao1, Chao2, Jackknife 1, Jackknife 2, Bootstrap, MMMean), 2) calculated the estimated mean of sample coverage, 3) chose the most accurate estimator for sample coverage according to the tables provided by Brose and Martinez [40], and 4) estimated species richness with this estimator and with the maximum number of SOCs per forest type.

For the calculation of alpha and beta diversity we adopted the framework of Jost [10], [11] for partitioning gamma diversity into its alpha and beta component according to the formula: H_α_ * H_β_ = H_γ_ (H = Shannon Wiener entropy). Entropies, like the Shannon-Wiener index, are not themselves diversities, and their use may obscure differences in diversity because indices differ only by small magnitudes. For this reason we used the effective number of species (D) introduced by Jost [10] as a measure of “true diversity”, which was calculated from Shannon entropy (H) according to the formula in Jost [10] as D = exp (H). The effective species number (Hill number) equals species richness for the case that all species of a sample have the same frequency. Beta-diversity of N communities was calculated according to Jost [10] with H_ß = _1.0 representing N totally equal communities and H_ß = _N representing N completely different communities.

Partitioning of plain diversity can be biased if we do not account for unseen species. Alpha Shannon H_α_ entropy is an index that is sensitive to under-sampling and may lead to biased results. Chao and Shen [2] developed an unbiased estimator *H_α_ for the estimation of the index. Beck and Schwanghart [4] have proven that the ‘effective number of species’ based on the bias-corrected Shannon entropy *H_α_ is an unbiased estimator of diversity at sample completeness *c*. >0.5. Estimates of diversity from samples with sample completeness below this value are still less biased than estimated species richness calculated after Brose [40].

Marcon *et al.* [19] developed an unbiased estimator for Shannon beta-entropy, *H_β_, which we used in our study and which also works well at *c*. >0.5 sample completeness. *H_β_ is derived from an independently calculated *H*_β_, which is developed in same the paper [19]. Unbiased values are estimated separately for alpha, beta, and gamma diversity from the observed data. As there is no mathematical correction so that entropy sums up with no error, the sum of unbiased entropies, *H_α_ +*H_β_ = *Hγ, contains slight error [19]. Unbiased estimated entropies can be converted to true community numbers, with *D_β_ ranging from 1, indicating a perfect equality of distribution and species composition, to N, indicating equal numbers of samples (n = N) with no species in common.

We also needed a correction for differing sample size. Unequal community samples do not mainly arise because of unequal sampling effort (e.g. comparing 20 and 30 Winkler sampling points), but are a permanent problem in all individual based diversity calculations, as no method guarantees similar individual numbers in the samples to be compared. Thus either weighting of the results according to community abundances or standardization on similar individual numbers is necessary [3].

We therefore performed basic calculation of alpha diversity indices at a common sample size of 206 SOCs for all four forests, and a second time at 470 SOCs only for the alluvial and limestone forests. We also calculated Simpson diversity at 206 SOCs as a diversity measure of order 2 [11].

For diversity partitioning we weighted the plots according to sample size. To explore significant deviations of observed alpha and beta components of diversity from those expected by chance we partitioned species richness and diversity across hierarchical scales and compared these results with the results of a random distribution [41].

For the calculations of the biased corrected alpha, beta and gamma-diversity indices and their transformation into true diversities we used the R [42] code provided by [19]. These calculations were made with the full samples size for each forest type and accounted for the respective differences.

To assess the geographical distribution of beta diversity within and among forest types, we estimated distances between the mid-points of each of the six transects as well as between all single sampling points with Google Earth^©^ and wrote them to distance matrices. Some of these single transects had a sample completeness *c*. <0.5, thus restraining us from calculating *H_β_ as we did with the sample point results. Instead we calculated the unbiased estimated Chao-Sørensen Index that includes unseen species [9], as well as the Morisita-Horn Index as an order 2 measure of beta diversity, which gives more weight to the most abundant species and is thus less biased by singletons and unseen species [5]. Both similarity indices were converted to dissimilarities by subtracting them from 1 to allow processing with a mantel test, which was used to compare the resulting data matrices with a distance matrix of the localities and was calculated with 999 randomizations [43]. Similarly we compared Chao-Sørensen Indices and distances for all 100 Winkler sampling points.

## Supporting Information

Table S1 List of ant species and their numbers of occurrences in the four forest types.
(XLSX)

Table S2 Unbiased estimations of alpha, beta and gamma Shannon diversity index *H according to the partitioning procedure of Marcon *et al.* [19].
(PDF)
