# Supplementary material for: Biodiversity Assessment in Incomplete Inventories: Leaf Litter Ant Communities in Several Types of Bornean Rain Forest
Source: PLoS One. 2012 Jul 16;7(7):e40729. doi: 10.1371/journal.pone.0040729 (PMC3398027; doi:10.1371/journal.pone.0040729)
Supplement: Table S2 — Unbiased estimations of alpha, beta and gamma Shannon diversity index *H according to the partitioning procedure of Marcon et al. [19]. (PDF) [file pone.0040729.s002.pdf]

Table S2. Unbiased estimations of alpha, beta and gamma Shannon diversity index $^*H$ according to the partitioning procedure of Marcon *et al.* (2011).

Diversity measurement	Alluvial	Limestone	Dipterocarp	Kerangas	Weighted sum
Unbiased alpha Shannon diversity index $^*H_\alpha$	4.367	4.471	4.264	4.084	4.350
Unbiased beta Shannon diversity index $^*H_\beta$	0.321	0.238	0.450	0.611	0.352
					[0.319; 0.387]
Unbiased Shannon gamma diversity index $^*H_\gamma$					4.705

Given are the unbiased estimators for the alpha and beta Shannon diversity index, for the single forest types and their weighted sums, as well as the resulting unbiased gamma Shannon diversity index. Upper and lower 95% confidence interval for the estimator of Shannon beta diversity are shown in squared brackets.

Marcon E, Hérault B, Baraloto C, Lang G (2011) The decomposition of Shannon's entropy and a test for beta diversity. Oikos 121: 516-522.
